# Nasopharyngeal carcinoma with unusual metastatic spread to the spine and meninges: a case report with literature review

**DOI:** 10.1093/jscr/rjaf022

**Published:** 2025-01-23

**Authors:** Salma Swadi Yassen, Sajjad Ghanim Al-Badri, Ali Naser Aldarawsha, Mohamed Samy Elazab, Asdah Alawad, Ameer Dhahir Hameedi, Abdulrahman Khaldoon Hamid, Hussein Mohsin Hasan, Nabeel Al-Fatlawi, Hasan Ali Asghar

**Affiliations:** Warith International Cancer Institute, Karbala, Iraq; College of Medicine, University of Baghdad, Baghdad, Iraq; Warith International Cancer Institute, Karbala, Iraq; Warith International Cancer Institute, Karbala, Iraq; Warith International Cancer Institute, Karbala, Iraq; Warith International Cancer Institute, Karbala, Iraq; Alexandria University College of Medicine, Alexandria, Egypt; College of Medicine, University of Baghdad, Baghdad, Iraq; College of Medicine, University of Baghdad, Baghdad, Iraq; Warith International Cancer Institute, Karbala, Iraq

**Keywords:** nasopharyngeal carcinoma (NPC), central nervous system metastases, spinal metastases, Epstein–Barr virus (EBV), aggressive metastatic behavior

## Abstract

Nasopharyngeal carcinoma (NPC) is an epithelial malignancy commonly associated with Epstein–Barr virus infection. While bone, liver, and lung metastases are well-documented, central nervous system (CNS) involvement, particularly spinal and meningeal metastases, is extremely rare. We present a 41-year-old male with nasal obstruction and diplopia, diagnosed with locally advanced NPC. After treatment with chemotherapy and intensity-modulated radiotherapy, the patient achieved excellent locoregional control. However, months later, he developed persistent back pain, and imaging revealed metastatic deposits in the spine and meninges. Histopathological analysis confirmed metastatic NPC despite resolution of the primary tumor. The patient received palliative radiotherapy and intrathecal chemotherapy, but disease progression highlighted the aggressive nature and poor prognosis of CNS metastases in NPC. This case underscores the need for advanced imaging, histological confirmation, and tailored therapies in managing rare NPC metastases, with long-term follow-up and innovative therapies critical for improving outcomes in advanced disease.

## Introduction

Nasopharyngeal carcinoma (NPC) is a rare malignancy that originates in the epithelial lining of the nasopharynx. It is most prevalent in certain geographic regions, including Southern China, Southeast Asia, and North Africa, while being relatively uncommon in Western countries [1]. NPC is unique among head and neck cancers due to its strong association with Epstein–Barr virus (EBV) infection, particularly in endemic areas [2]. The disease often presents with nonspecific symptoms such as nasal obstruction, epistaxis, or neck masses, which can lead to delayed diagnosis.

The metastatic behavior of NPC is notably aggressive, with common distant sites including the bones, lungs, and liver. Central nervous system (CNS) involvement, however, is exceedingly rare and poses significant diagnostic and therapeutic challenges [3]. When NPC metastasizes to the brain, meninges, or spinal cord, it often signifies advanced disease with limited treatment options. This pattern of spread typically occurs via hematogenous routes or direct extension through the skull base [4].

Advancements in imaging and treatment modalities, such as intensity-modulated radiotherapy (IMRT) and cisplatin-based chemotherapy, have improved locoregional control of NPC. However, distant metastases remain a critical challenge in disease management. Rare metastatic presentations, including CNS involvement, underscore the need for vigilant monitoring, multidisciplinary care, and innovative therapeutic strategies to improve outcomes for affected patients. This case highlights the complexity of managing NPC with unusual metastatic spread to the spine and meninges, emphasizing the importance of tailored treatment approaches in addressing this aggressive and multifaceted disease.

## Case presentation

A 41-year-old man presented with progressive nasal obstruction and diplopia, with no history of pain, nasal bleeding, or systemic symptoms such as fever, weight loss, or fatigue. Physical examination revealed fullness in the nasopharyngeal region and restricted lateral gaze in one eye. Imaging studies, prompted by these symptoms, identified a nasopharyngeal mass. Biopsy confirmed nonkeratinizing undifferentiated nasopharyngeal carcinoma. Staging positron emission tomography-computed tomography (PET-CT) showed a locally advanced mass invading the skull base and metastatic involvement of bilateral retropharyngeal and cervical lymph nodes, along with concerning findings in the right humerus and liver.

**Figure 1:**
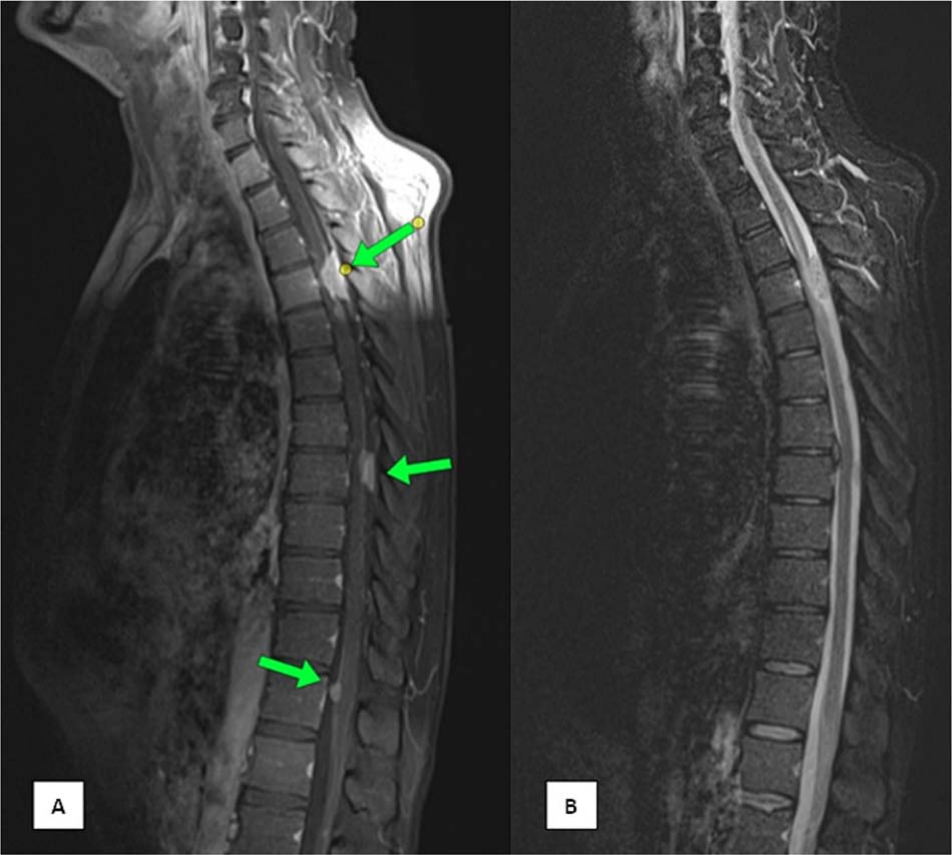
MRI of the spine (A) sagittal T1-weighted fat-saturated post-contrast image and (B) sagittal Short Tau Inversion Recovery (STIR) sequence demonstrating multiple intra-spinal, extra-medullary meningeal-based enhancing lesions. The largest lesion measures 22 × 11 mm at the D4 level.

The patient underwent three cycles of chemotherapy, achieving near-complete metabolic resolution of the nasopharyngeal mass, regression of lymph nodes, and resolution of liver and humeral lesions. Subsequently, he completed 33 fractions of radiotherapy. Post-treatment PET-CT showed no recurrence but revealed focal increased fluorodeoxyglucose (FDG) uptake at spinal cord levels T4 and T8, necessitating magnetic resonance imaging (MRI) evaluation. MRI identified dorsal intraspinal enhancing soft tissue lesions indenting the spinal cord (Fig. 1), consistent with dural-based metastases.

**Figure 2:**
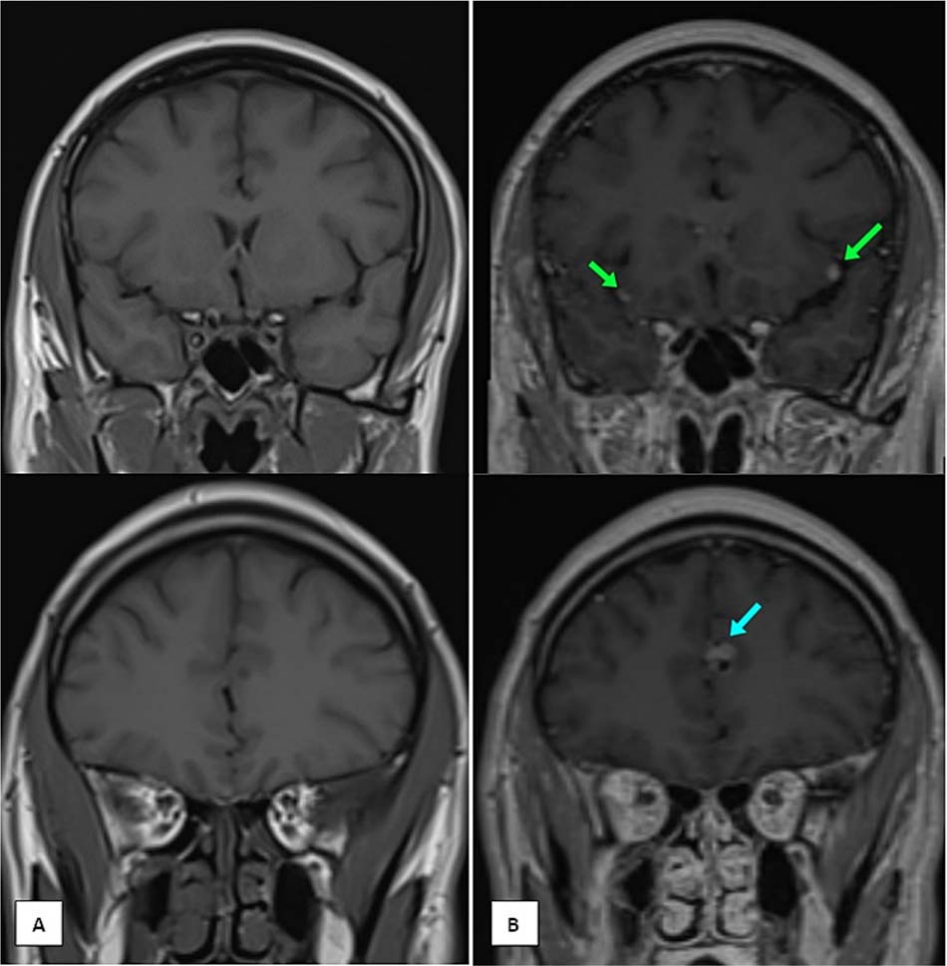
MRI of the brain (A) coronal T1 pre-contrast and (B) coronal T1 post-contrast images showing multiple leptomeningeal enhancing nodules. The largest are seen at the left frontal parafalcine region (as shown in the single arrow) and bilateral sylvian fissures (as shown in the two arrows).

**Figure 3:**
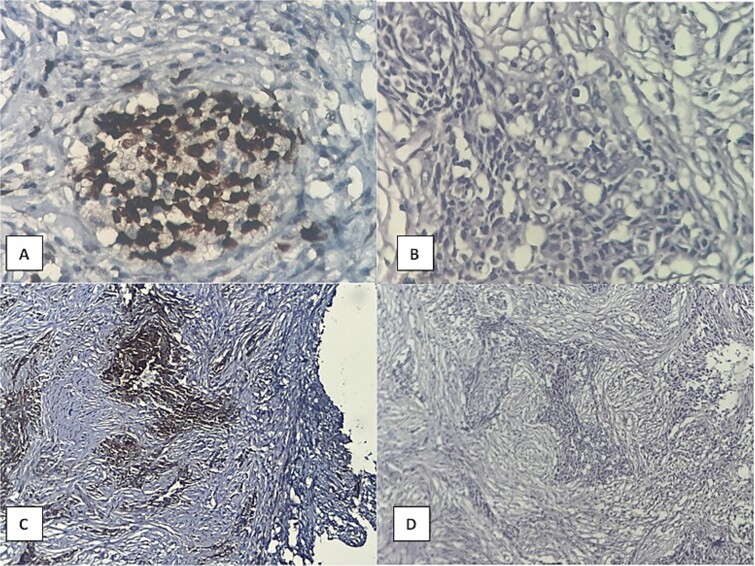
Histopathological examination showing (A) prominent PD-L1 expression, (B) high power of tumor cell infiltration, (C) P63 expression, (D) malignant epithelial cell infiltration of desmoplastic stroma.

Months later, persistent back pain led to further imaging, which revealed progressive intradural lesions and new extra-axial meningeal nodular lesions likely representing meningeal deposits (Fig. 2). Despite the absence of recurrent nasopharyngeal mass, biopsy of the spinal lesions confirmed metastatic NPC (Fig. 3), indicating disease progression despite an initial excellent response to treatment.

The patient’s case highlights the complexity of managing NPC with advanced metastatic disease. He is receiving palliative care to manage symptoms and prevent further progression, with close follow-up and multidisciplinary care essential to his ongoing management.

## Discussion

NPC is a tumor originating from the nasopharynx, typically metastasizing to bones, liver, and lungs [5, 6]. However, metastases to the CNS, such as the spine and meninges, are exceedingly rare. This case highlights the importance of recognizing atypical presentations of NPC and maintaining a high level of suspicion for CNS involvement.

NPC is prevalent in specific regions, including Southern China, Southeast Asia, and North Africa [7]. EBV, strongly associated with NPC in these endemic areas, contributes to its development through mechanisms such as inducing genetic instability, immune suppression, and cellular transformation [8]. Understanding the molecular pathways linking EBV to distant metastasis in NPC warrants further research.

CNS metastases in NPC are primarily thought to occur via hematogenous spread through the venous plexus [6], although other routes, such as direct extension through the skull base and lymphatic dissemination, cannot be excluded [9]. In this case, hematogenous spread appears likely, as metastases to the spine and meninges occurred despite a complete response to chemotherapy, indicating aggressive disease behavior and potential for late recurrence.

Diagnosing CNS metastases in NPC is challenging due to their rarity and atypical symptoms. MRI and PET-CT are valuable tools for identifying metastatic progression. MRI effectively detects dural-based and meningeal lesions, while PET-CT provides metabolic evidence of malignancy [10, 11]. However, a biopsy remains essential for definitive diagnosis.

This case demonstrates the unique metastatic pattern of NPC even after treatment, highlighting the importance of advanced imaging and long-term follow-up. Persistent symptoms, such as back pain, should prompt further investigation to rule out metastatic progression.

The management of CNS metastases in NPC is complex and challenging. Standard approaches, including intrathecal cisplatin-based chemotherapy and IMRT, may provide temporary symptom relief but often fail to prevent disease progression [12]. Emerging therapies, such as agents targeting EGFR and PD-1/PD-L1 pathways, offer potential but require further evaluation for efficacy in CNS involvement [13].

CNS metastases in NPC signify advanced disease with a poor prognosis, underscoring the need for comprehensive imaging and follow-up in high-risk patients. While this case provides valuable insights, it is limited by its single-case nature, lack of genetic analysis, and inability to generalize findings [14]. Future research should focus on genetic analysis, predictive biomarkers for metastasis, and advanced imaging techniques to improve early detection and treatment outcomes [15].

## Conclusion

This case underscores the rare and aggressive nature of NPC metastasizing to the spine and meninges, highlighting significant diagnostic and therapeutic challenges. Despite excellent locoregional control, CNS metastases demonstrate the potential for late progression, emphasizing the need for long-term follow-up, advanced imaging, and histopathological confirmation. The poor prognosis associated with CNS involvement calls for innovative systemic therapies targeting specific molecular pathways. Multidisciplinary care remains essential in managing the complexities of such advanced cases and improving patient outcomes.
